# Transient sinus arrest caused by coronary spasm of the sinus node artery after pulsed field ablation for superior vena cava isolation: a case report

**DOI:** 10.1093/ehjcr/ytag703

**Published:** 2026-09-21

**Authors:** Rintaro Hojo, Koichiro Yamaoka, Tomoyuki Arai, Masao Takahashi, Seiji Fukamizu

**Affiliations:** Department of Cardiology, Tokyo Metropolitan Hiroo Hospital, 2-34-10 Ebisu, Shibuya-ku, Tokyo 150-0013, Japan; Department of Cardiology, Tokyo Metropolitan Hiroo Hospital, 2-34-10 Ebisu, Shibuya-ku, Tokyo 150-0013, Japan; Department of Cardiology, Tokyo Metropolitan Hiroo Hospital, 2-34-10 Ebisu, Shibuya-ku, Tokyo 150-0013, Japan; Department of Cardiology, Tokyo Metropolitan Hiroo Hospital, 2-34-10 Ebisu, Shibuya-ku, Tokyo 150-0013, Japan; Department of Cardiology, Tokyo Metropolitan Hiroo Hospital, 2-34-10 Ebisu, Shibuya-ku, Tokyo 150-0013, Japan

**Keywords:** Pulsed field ablation, Sinus arrest, Sinus node artery, Coronary spasm, Superior vena cava isolation, Case report

## Abstract

**Background:**

Pulsed field ablation (PFA) has emerged as a novel non-thermal ablation modality with preferential myocardial selectivity. However, its effects on adjacent structures, including the sinus node and its arterial supply, remain incompletely understood.

**Case summary:**

A 72-year-old woman underwent repeat ablation for recurrent paroxysmal atrial fibrillation. Following pulmonary vein isolation, superior vena cava (SVC) isolation was performed using a PFA system. Sinus rhythm was initially preserved. However, sinus arrest developed several minutes after ablation. High right atrial backup pacing was initiated. Coronary angiography revealed severe spasm of the sinus node artery. Sinus node function gradually recovered after supportive management. Post-procedural voltage mapping confirmed successful SVC isolation.

**Discussion:**

This case highlights delayed sinus arrest following SVC isolation using PFA, with angiographic evidence suggesting sinus node artery spasm. While multiple mechanisms may contribute to sinus node dysfunction after PFA, coronary vasospasm should be considered, particularly in cases with delayed onset.

Learning points

- Pulsed field ablation (PFA) for superior vena cava (SVC) isolation may induce coronary artery spasm of the sinus node artery, leading to transient sinus arrest.
- When delayed and persistent sinus arrest occurs after SVC isolation using PFA, spasm of the sinus node artery may be considered as a possible mechanism.
- Coronary artery anatomy of the sinus node should be considered when delivering PFA near the right atrium–SVC junction, and the potential role of prophylactic nitrate administration warrants further investigation.

## Introduction

Superior vena cava (SVC) isolation using pulsed field ablation (PFA) has been reported to be effective and relatively safe,^1,2^ although transient sinus node (SN) dysfunction has occasionally been observed immediately after PFA application.^1^ In addition, transient coronary artery spasm induced by PFA applied near the coronary arteries has been reported.^3,4^ We report a case of delayed sinus arrest after SVC isolation using PFA, highlighting potential multifactorial mechanisms including sinus node artery (SNA) spasm.

## Summary figure

**Figure:**
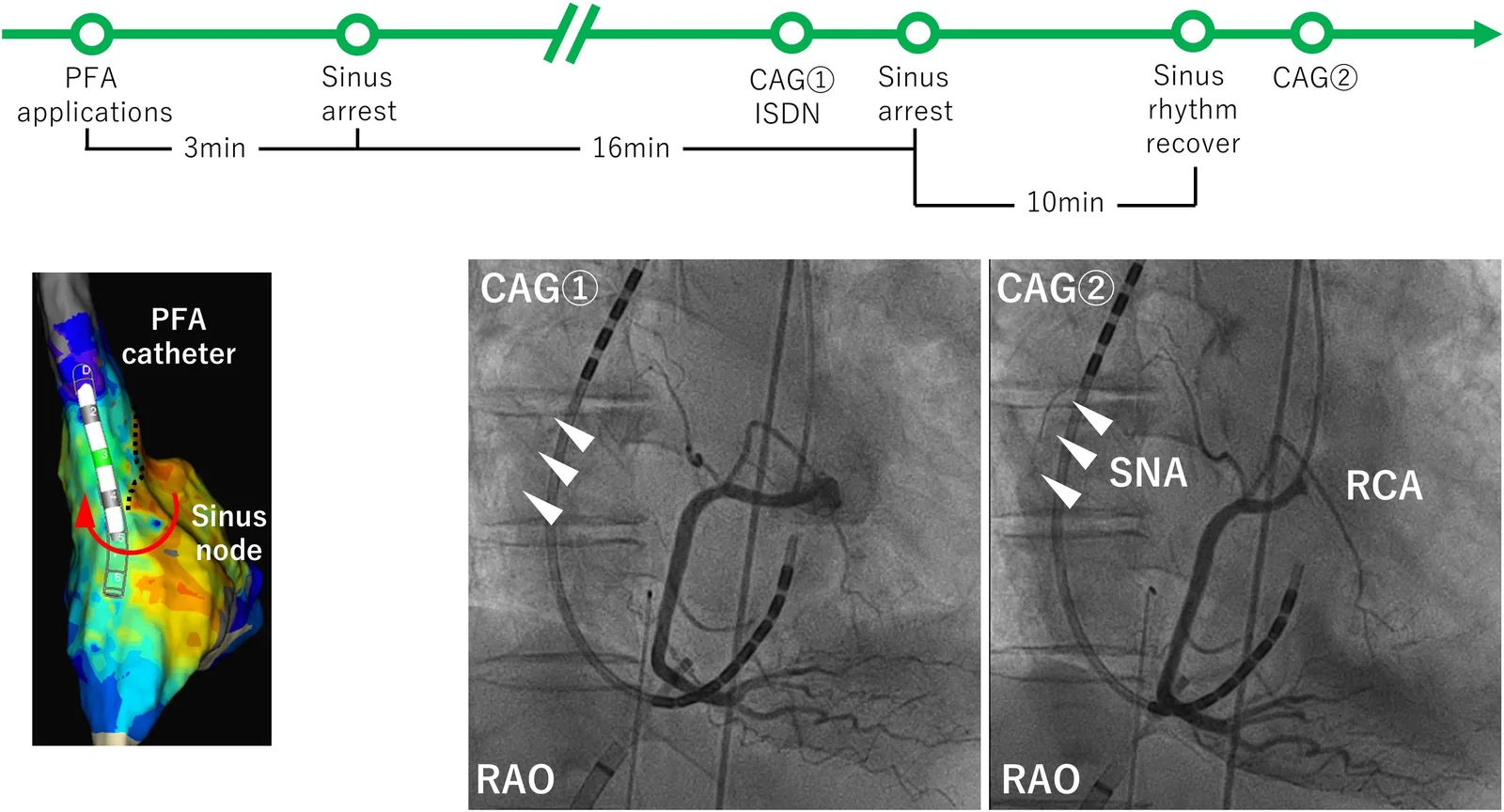


## Case presentation

A 72-year-old woman with paroxysmal atrial fibrillation underwent pulmonary vein isolation using PFA. During the index procedure, no non-pulmonary vein triggers were identified. Atrial fibrillation recurred 6 months later, and a second ablation procedure was performed after obtaining informed consent. The patients received therapeutic anticoagulation for ≥4 weeks, and intravenous heparin was administered to maintain an activated clotting time > 300 s during the procedure. Electroanatomical mapping was performed using the EnSite X system (Abbott, Abbott Park, IL, USA). At the repeat procedure, no spontaneous or provoked SVC firing was observed. However, given the patient’s recurrent paroxysmal atrial fibrillation after durable pulmonary vein isolation, empirical SVC isolation was performed based on the operator’s discretion. Ablation was conducted using a circular array PFA catheter (PulseSelect™, Medtronic, Minneapolis, MN, USA).

Prior to SVC isolation, a detailed activation map of the right atrium–SVC junction was created during sinus rhythm using a multipolar mapping catheter (Advisor™ HD Grid, Abbott, Abbott Park, IL, USA) to identify the location of the SN and spontaneous conduction block lines. The activation map demonstrated a conduction block line located just superior to the SN, and conduction between the right atrium (RA) and SVC was observed through the posterior wall of the left atrium (*Figure 1*). Based on these findings, PFA was applied to this conduction pathway. The circular array PFA catheter was partially exposed from the sheath up to the fifth electrode, and PFA was delivered while carefully avoiding the SN region identified on activation mapping. A standard application consists of four biphasic pulses (4 PFA). However, in this case, each pulse was delivered individually in manual mode while continuously monitoring SN function, and the subsequent pulse was delivered only after confirming the absence of SN dysfunction. A total of four applications were delivered.

**Figure 1:**
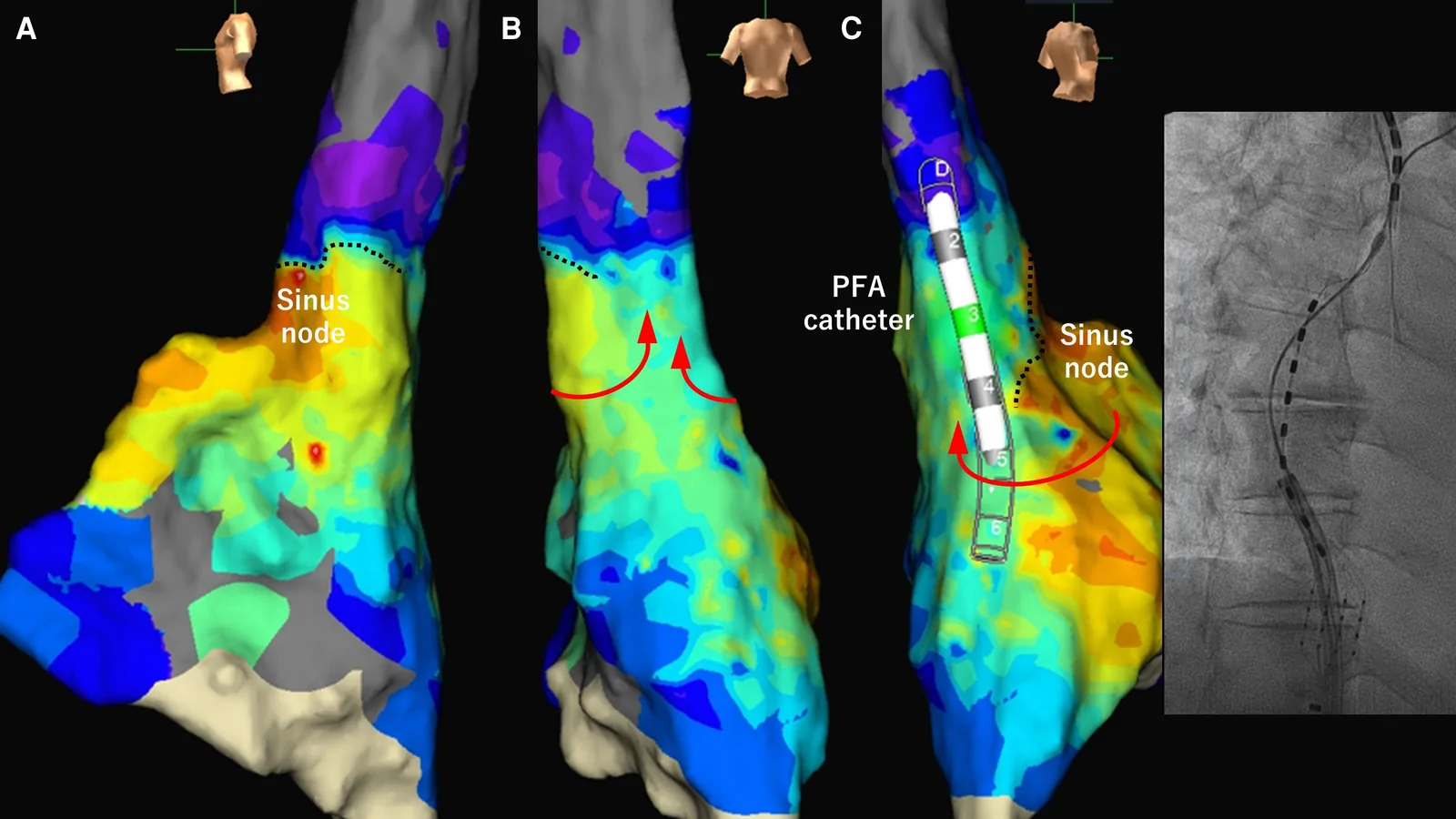
Activation mapping and ablation site at the right atrium–superior vena cava junction. (*A*) Activation map during sinus rhythm showing the location of the sinus node (SN) and a spontaneous conduction block line (dotted line) just superior to the SN. (*B*) Posterior view demonstrating preserved conduction between the right atrium (RA) and superior vena cava (SVC) through the posterior pathway (red arrows), bypassing the spontaneous conduction block line. (*C*) Fluoroscopic and electroanatomical mapping view showing the circular pulsed field ablation (PFA) catheter positioned along the posterior RA–SVC junction targeting the remaining conduction pathway.

Immediately after the applications, sinus rhythm with a heart rate of 50–60 b.p.m. was observed. Sinus arrest occurred ∼1 min later and backup pacing from the high RA was initiated. Although temporary pacing was briefly discontinued to assess intrinsic sinus rhythm, sinus arrest persisted. Because coronary artery spasm was suspected, emergent coronary angiography (CAG) was performed, which revealed complete interruption of blood flow in the SNA originating from the right coronary artery (RCA) (*Figure 2*, CAG ①). Left CAG demonstrated no SNA arising from the left circumflex artery.

**Figure 2:**
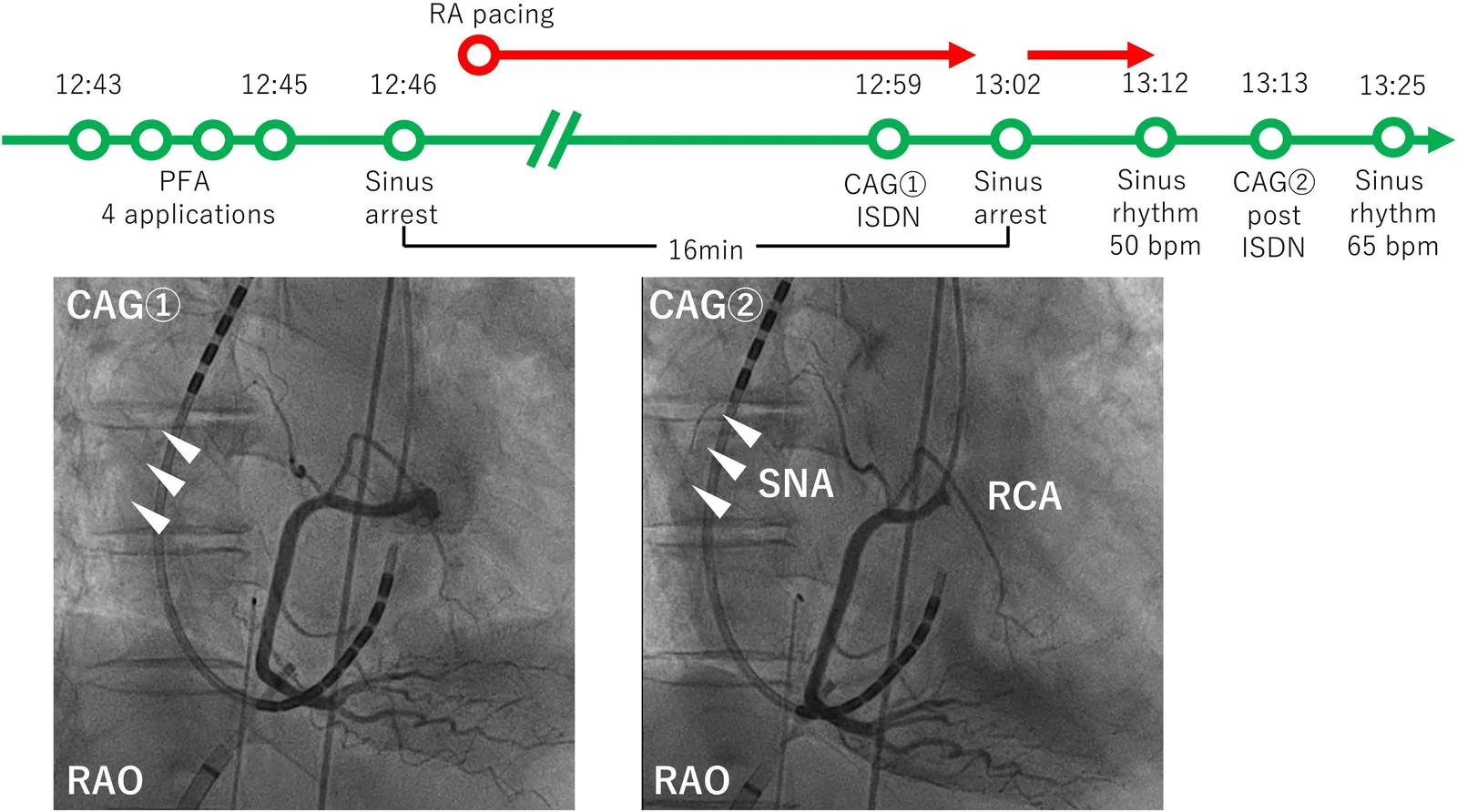
Timeline of sinus arrest and coronary angiographic findings after pulsed field ablation. A structured timeline of events is shown. After four pulsed field ablation applications, sinus arrest occurred ∼1 min later, and high right atrial pacing was initiated. Coronary angiography (CAG ①) revealed complete interruption of blood flow in the sinus node artery (arrowheads) arising from the right coronary artery. Following CAG ① intracoronary, isosorbide dinitrate was administered into the right coronary artery. Sinus rhythm resumed 26 min after onset. Repeat angiography (CAG ②) demonstrated restoration of blood flow in the sinus node artery. Stable sinus rhythm was subsequently maintained.

Sinus arrest persisted for 16 min. Following the initial CAG, a bolus of intracoronary isosorbide dinitrate was administered into the RCA. Sinus rhythm resumed at 50 b.p.m. 26 min after the onset of sinus arrest. Repeat CAG demonstrated restoration of blood flow in the SNA (*Figure 2*, CAG ②). Ultimately, stable sinus rhythm at 65 b.p.m. was achieved. A voltage map was created during sinus rhythm after the procedure, confirming electrical isolation of the SVC.

At 4 months of follow-up, the patient remained in stable sinus rhythm without recurrence of atrial fibrillation or SN dysfunction.

## Discussion

Sinus node dysfunction is a well-recognized complication of SVC isolation using thermal ablation techniques.

Delayed SN dysfunction after ablation has been reported, suggesting that mechanisms beyond direct thermal injury and autonomic effect, such as tissue oedema and SNA injury, may contribute. However, the mechanisms of SN dysfunction following PFA remain less well defined.

Chen *et al*. previously reported a case of prolonged sinus arrest with a sustained junctional escape rhythm lasting ∼30 min after SVC isolation using PFA, highlighting the potential vulnerability of the SN during SVC ablation.^5^ They discussed direct SN injury and autonomic effects as possible mechanisms.

In this case, PFA was delivered near the SN, and direct injury to the SN by PFA cannot be completely excluded. Although the relative contribution of direct SN injury and transient ischaemia due to SNA spasm cannot be determined in this single case, the delayed onset of sinus arrest, ∼2 min after the initial PFA application and 1 min after the final application, together with angiographic evidence of transient SNA occlusion, suggests that transient ischaemia due to coronary vasospasm likely played a major role in the development of SN dysfunction.

The SN is supplied by branches arising from either the RCA, the left circumflex artery, or both.^6^ In cases where the SNA arises solely from the left circumflex artery, SN dysfunction has been reported after radiofrequency ablation of the left atrial roof due to injury of the arterial branch supplying the SN.^7^

In the present case, the SNA originated exclusively from the RCA and coursed along the posterior aspect of the SVC towards the SN. We speculate that PFA may have induced coronary spasm of the SNA, resulting in ischaemia of the SN and delayed onset of sinus arrest after PFA application.

Recent studies have demonstrated that PFA can induce coronary vasospasm, particularly when delivered near coronary arteries. Coronary artery spasm has been reported after PFA applied at the mitral isthmus and cavotricuspid isthmus,^3,4^ affecting the left circumflex and RCA, respectively. Therefore, coronary spasm of the SNA may also occur during PFA delivery at the RA–SVC junction. Previous reports have suggested that PFA-induced coronary spasm can be prevented by nitrate administration.^4^ Thus, prophylactic intravenous administration of nitrates before or during PFA near the SNA may be considered as a potential strategy to mitigate the risk of SN dysfunction. However, its effectiveness, particularly in the microvascular circulation, remains uncertain and requires further investigation. For delayed and persistent sinus arrest, coronary vasospasm may be a possible underlying mechanism, and treatment with nitrates could be considered.

## Limitations

First, the possibility that SN dysfunction was caused by mechanisms other than SNA injury cannot be excluded. Finally, although SVC isolation using PFA currently remains an off-label application, the development of more focal and selective PFA technologies may expand its potential role in the future.

## Conclusion

This case demonstrates that transient sinus arrest may occur due to coronary spasm of the SNA induced by PFA during SVC isolation. Preventive strategies, including prophylactic nitrate administration, may warrant further investigation before PFA near the SVC to reduce the risk of coronary spasm and subsequent SN dysfunction.

## Lead author biography


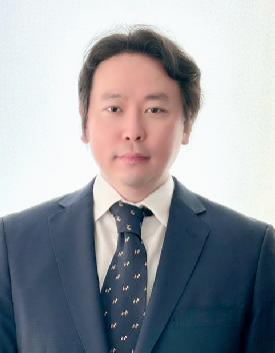


Dr Hojo received an MD degree from the University of Chiba School of Medicine, Chiba, Japan, in 2005, and completed 2 years of Japanese general residency training at the Japanese Red Cross Medical Center. Since 2007, Dr Hojo has been working as a cardiologist at Tokyo Metropolitan Hiroo Hospital.

**Consent:** Written informed consent for publication of this case report and the accompanying images was obtained from the patient in accordance with the COPE guidelines.

## Data Availability

The data underlying this article are available within the article. Additional data are available from the corresponding author upon reasonable request.
